# Percutaneous Valve-in-Valve Treatment of a (Very Old and Fluoroscopy Invisible) Degenerated Tricuspid Prosthesis Through the Right Jugular Vein Approach

**DOI:** 10.3389/fcvm.2019.00022

**Published:** 2019-03-19

**Authors:** Cristina Aurigemma, Francesco Burzotta, Michele Corrado, Christian Colizzi, Carlo Trani

**Affiliations:** ^1^Institute of Cardiology, Fondazione Policlinico Universitario A. Gemelli IRCSS, Rome, Italy; ^2^Università Cattolica del Sacro Cuore, Rome, Italy

**Keywords:** tricuspid valve, degenerated prosthesis, percutaneous valve in valve, tricuspid regurgitation, tricuspid stenosis

## Abstract

Tricuspid valve dysfunction adversely affects prognosis and may cause severe symptoms. Among the different opportunity offered by transcatheter techniques, the valve in valve represents an emerging strategy to treat patients with degenerated surgical biological prosthesis. We describe a case report of a percutaneous valve in valve treatment of a very old and fluoroscopy invisible tricuspid degenerated bioprosthesis. In the reported case, pivotal issue for percutaneous valve in valve procedure success was the achievement of perfect alignment between transcatheter valve and degenerated bioprosthesis despite the horizontal right chamber axis and the poor valve visibility. Of note, the combination of jugular vein approach, transapical delivery system rotation, right ventricle guidewire placement, and right atrium angiography made the valve in valve procedure safely.

A 71 years old woman was admitted to our hospital due to worsening congestive heart failure. Her long-lasting clinical history was characterized by rheumatic valve disease previously treated three times by cardiac surgery (1975: mitral valvuloplasty; 1982: mitral and tricuspid valve replacement with Liotta porcine bioprosthesis, 28 and 30 respectively; 1994: mitral valve replacement with mechanical valve and epicardial pacemaker implantation).

Echocardiographic examination showed tricuspid prosthesis degeneration with both severe stenosis and regurgitation (Figure 1). The right heart chambers were dilated and right ventricular dysfunction was present while normal left ventricular function and normal mitral mechanical valve function was documented. In heart-team discussion, cardiac surgery was ruled out due to prohibitive operative risk and compassionate treatment by attempt for percutaneous tricuspid valve in valve treatment was proposed as the most reliable option. After ethics committee approval and patient's written consent obtainment, the procedure was planned in our hybrid room. Of note, the tricuspid Liotta prosthesis was not visible at fluoroscopy. Trans-jugular access offers a good angle to approach the tricuspid valve, with a delivery system that requires less steering, but requires a vein large enough to accommodate the sheath without being damaged. The femoral vein is the safest access route due to its size, but the angle between the inferior vena cava and tricuspid valve is steep and may hamper the procedure. In our case the transcatheter tricuspid valve replacement was a valve in valve procedures and the dimension of the tricuspid bioprosthesis annulus was not large. Therefore, a right jugular vein was selected as the primary access (in order to facilitate coaxiality with the tricuspid prosthesis). A left jugular vein was selected as an additional access (in order to monitor right side pressures and administer drugs) and transesophageal echocardiography (TEE) monitoring was adopted to guide the procedure.

**Figure 1:**
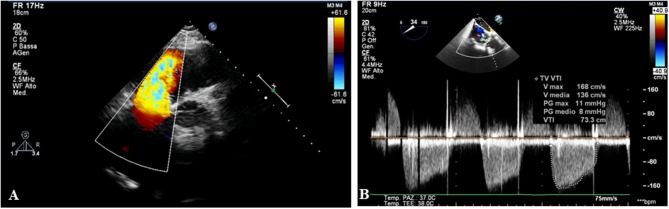
The echocardiography shows a degeneration of tricuspid biological prosthesis with severe regurgitation **(A)** and stenosis **(B)**.

The tricuspid degenerated prosthesis was successful crossed by a Swan Ganz catheter with an angiographic 0.035' wire. Then, the angiographic wire was exchanged with a superstiff 0.035' wire (placed into the pulmonary artery system) which allowed to safely perform balloon valvuloplasty (using the 23 mm Edwards Balloon), (Figure 2). Even if pre-dilatation of the prosthesis is not mandatory in valve in valve procedures, we have decided to perform a balloon valvuloplasty to confirm the bioprosthesis valve size and to visualize a fluoroscopy invisible bioprosthesis. Then, on the bases of Liotta prosthesis internal measures, which was 30 mm in size, an Edwards Sapien 3 26 mm, a device normally used for aortic valve replacement, was selected to be implanted through the right internal jugular access using the 18F transapical delivery system. The transapical delivery system has been chosen because of its rotation system which could easily allow a better alignment with the degenerated valve despite the horizontal right chamber axis. Since neither fluoroscopy nor TEE allowed for reliable visualization of Liotta prosthesis, we decided to inject contrast media through the left internal jugular access obtaining a right atrial angiography which allowed precisely to control the valve in valve deployment (Figure 2; Supplementary Video 1). The TEE post procedure showed no regurgitation and no stenosis of the tricuspid valve (Figure 3). Post-intervention clinical course was uneventful and, on the seventh post-operative day, the patient was discharged.

**Figure 2:**
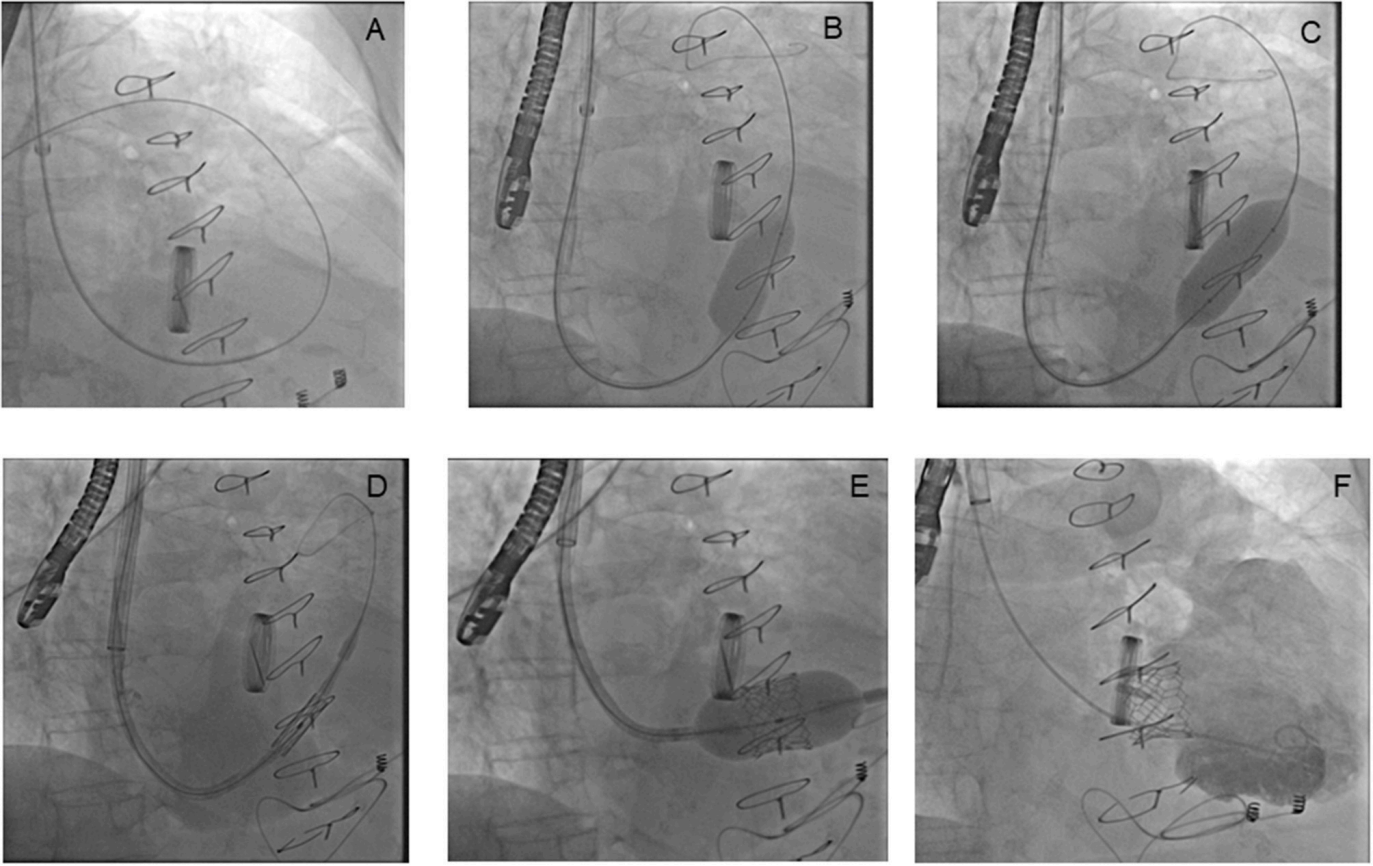
The implantation of an aortic balloon expandable biological valve into a degenerated tricuspid biological prosthesis. A Swan Ganz catheter with an angiographic 0.035' wire crosses the tricuspid degenerated prosthesis **(A)**. A balloon valvuloplasty is performed, even if no mandatory in valve in valve procedure. The first balloon inflation shows a residual stenosis **(B)**, the second inflation shows a successful balloon expansion. The valvuloplasty is also performed to confirm the degenerated bioprosthesis size **(C)**. An aortic balloon expandable biological valve, a device normally used for aortic valve replacement, crosses the tricuspid degenerated prosthesis **(D)**. The guidewire is placed in right ventricle to achieve a perfect alignment between transcatheter valve and degenerated bioprosthesis despite the horizontal right chamber axis. The valve is deployed using a 3D transesophageal echocardiography (TEE) reconstruction and right atrial angiography to control the release **(E)**. The final right ventricular angiography shows no residual regurgitation after valve in valve implantation **(F)**.

**Figure 3:**
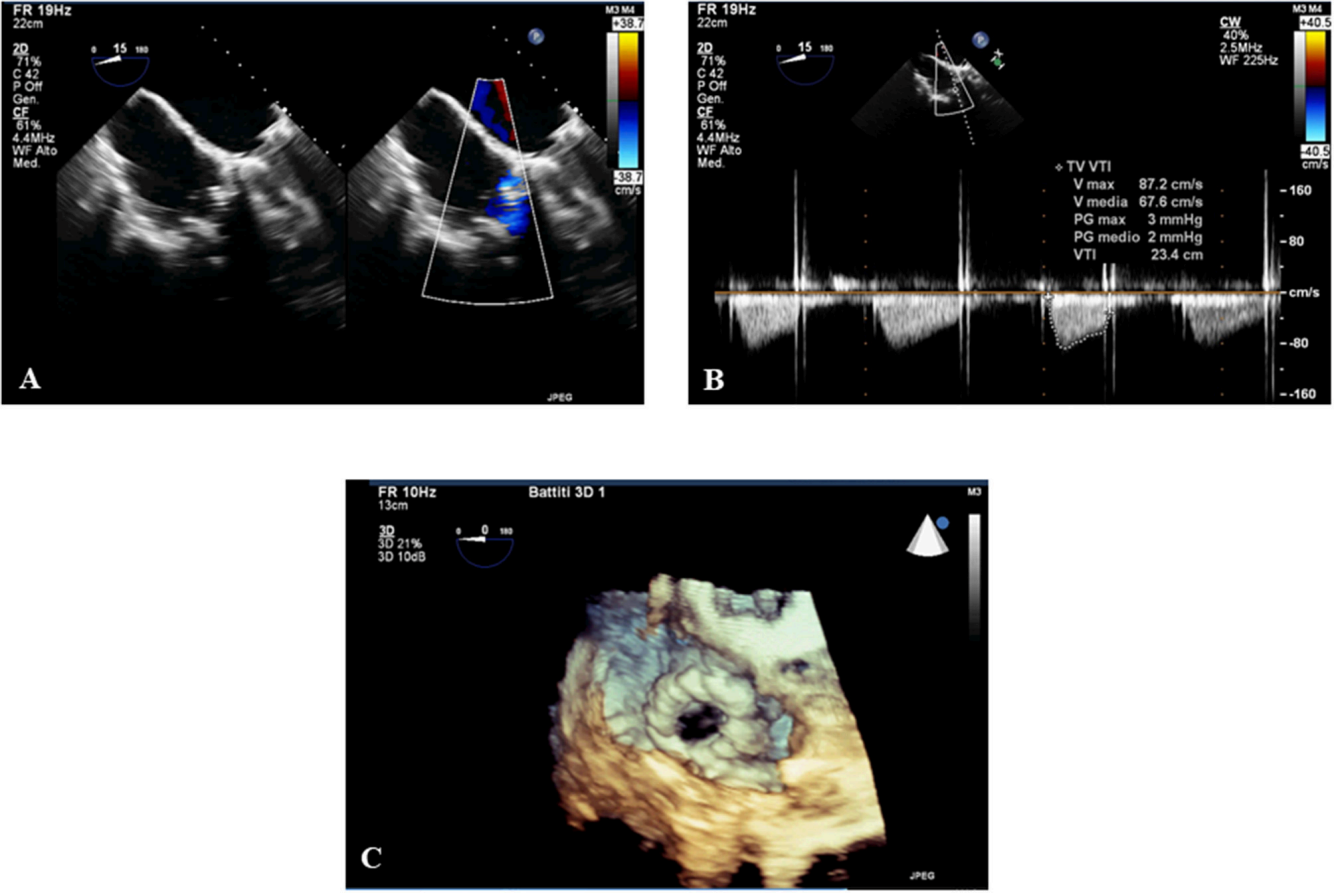
The 3D transesophageal echocardiography (TEE) post procedure shows the normal function of the tricuspid valve. The 3DTEE post procedure shows the absence of regurgitation **(A)**, stenosis **(B)**, and the 3D reconstruction **(C)** of the tricuspid prosthesis after the valve in valve intervention.

Tricuspid valve dysfunction adversely affects prognosis and may cause severe symptoms. Yet, due to high surgical risk, it often remains undertreated (1). Recently, the rapid development of trans-catheter approaches, allowed to considering tricuspid valve patients with high surgical risk as potential candidates for percutaneous approaches (2). Among the different opportunity offered by transcatheter techniques, the valve in valve represents an emerging strategy to treat patients with degenerated surgical biological prosthesis in the left system. Recently a sub analysis of VIVID registry data has demonstrated that TTVR was also hemodynamically and clinically beneficial in patients of various ages and underlying disease states. Indeed in 306 patients who underwent TTVR adverse valve-related outcomes were relatively uncommon, and valve function remained excellent in the vast majority of patients followed beyond 3 years post-TTVR (3).

In the present case, we faced a patient with severe, symptomatic, prosthesis degeneration of a rare valve implanted in tricuspid position. The Liotta valve was in the past adopted electively (by some surgeons) for the tricuspid replacement since its low profile allowed to minimize right ventricular cavity occupation (4). Yet, this porcine valve is uncommon and is not radiopaque thus making its percutaneous treatment particularly challenging. In the reported case, pivotal issue for procedure success was the achievement of perfect alignment between transcatheter valve and degenerated bioprosthesis despite the horizontal right chamber axis and the poor valve visibility. Of note, the combination of jugular vein approach, transapical delivery system rotation, right ventricle guidewire placement and right atrium angiography made the valve in valve procedure safely.

In conclusion, in the present case we have been able to successfully manage by valve-in-valve technique a degenerated tricuspid bioprostheses. The selection of appropriately sized aortic prosthesis and specific procedure adjustments may allow offer a percutaneous transcatheter treatment to patients with tricuspid prosthesis degeneration.

## Data Availability

All datasets generated for this study are included in the manuscript and/or the Supplementary Files.

## Author Contributions

All authors listed have made a substantial, direct and intellectual contribution to the work, and approved it for publication.

### Conflict of Interest Statement

FB discloses to have been involved in advisory board meetings or having received speaker's fees from Medtronic, St Jude Medical, Abiomed, Biotronic. CT discloses to have been involved in advisory board meetings or having received speaker's fees from St Jude Medical, Abiomed, Biotronic. CA discloses to have been involved in advisory board activities by Biotronic. The remaining authors declare that the research was conducted in the absence of any commercial or financial relationships that could be construed as a potential conflict of interest.

## Supplementary Material

Supplementary Video 1 A right atrial angiography is performed to control the release because of neither fluoroscopy nor TEE allow for reliable visualization of Liotta prosthesis.

## References

[1] Rogers JH Bolling SF. The tricuspid valve: current perspective and evolving management of tricuspid regurgitation. Circulation. (2009) 119:2718–25. 10.1161/CIRCULATIONAHA.108.842773 19470900

[2] Van Praet KM Stamm C Starck CT Sündermann S Meyer A Montagner M. An overview of surgical treatment modalities and emerging transcatheter interventions in the management of tricuspid valve regurgitation. Expert Rev Cardiovasc Ther. (2017) 16:75–89. 10.1080/14779072.2018.1421068 29283684

[3] McElhinney DB Aboulhosn JA Dvir D Whisenant B Zhang Y Eicken A Mid-term valve-related outcomes after transcatheter tricuspid valve-in-valve or valve-in-ring replacement. J Am Coll Cardiol. (2019) 73:148–57. 10.1016/j.jacc.2018.10.051 30654886

[4] Pavie A Bors V Piazza C Desruennes M Fontanel M Jault F. Mid-term results of the Liotta-Bioimplant low profile bioprostheses. J Card Surg. (1988) 3(Suppl. 3):353–8. 2980037 10.1111/jocs.1988.3.3s.353

